# Mechanical Properties and Fracture Behavior of a TC4 Titanium Alloy Sheet

**DOI:** 10.3390/ma15238589

**Published:** 2022-12-01

**Authors:** Zeling Zhao, Hongchao Ji, Yingzhuo Zhong, Chun Han, Xuefeng Tang

**Affiliations:** 1 College of Mechanical Engineering, North China University of Science and Technology, Tangshan 063210, China; 2 China 22 MCC Group Corporation Limited, Tangshan 063035, China; 3 State Key Laboratory of Materials Processing and Die & Mould Technology, Huazhong University of Science and Technology, 1037 Luoyu Road, Wuhan 430074, China

**Keywords:** TC4 titanium alloy, Johnson–Cook failure model, fracture behavior, plastic deformation

## Abstract

TC4 titanium alloy has excellent comprehensive properties. Due to its light weight, high specific strength, and good corrosion resistance, it is widely used in aerospace, military defense, and other fields. Given that titanium alloy components are often fractured by impact loads during service, studying the fracture behavior and damage mechanism of TC4 titanium alloy is of great significance. In this study, the Johnson–Cook failure model parameters of TC4 titanium alloy were obtained via tensile tests at room temperature. The mechanical behavior of TC4 titanium alloy during the tensile process was determined by simulating the sheet tensile process with the finite element software ABAQUS. The macroscopic and microscopic morphologies of tensile fracture were analyzed to study the deformation mechanism of the TC4 titanium alloy sheet. The results provide a theoretical basis for predicting the fracture behavior of TC4 titanium alloy under tensile stress.

## 1. Introduction

With the development of science and technology, the requirements for materials—especially metal materials—are increasing in various industrial fields. Titanium and titanium alloys are widely used in aerospace, military defense, and other high-tech fields due to their light weight, high specific strength, and good corrosion resistance [1,2,3]. Titanium alloy is mainly composed of α-phase, β-phase, and α + β two-phase titanium alloys; α + β two-phase titanium alloys are the most widely used, with good comprehensive performance and stable structure. TC4 is a typical two-phase titanium alloy [4]. Titanium alloy materials are prone to fracture failure under high-speed collision. Quasi-static tensile fracture is a typical failure mode of ductile metal materials [5]. The shape of metal materials changes under the action of external force. The process of material failure has three stages: elastic deformation, plastic deformation, and fracture failure [6,7,8]. The failure and damage of metal structures under complex stress states can occur during the tensile process, and strength failure is the most important failure behavior [9]. In strength failure, the influence of fracture failure is much greater than that of yield failure, so studying fracture failure is particularly important. Meanwhile, the classical strength theory is mainly applicable to the simple stress state of static loading, while the elastoplastic problem needs to consider the effect of average stress, which is not convenient to use. Therefore, establishing a set of fracture failure criteria suitable for complex stress states is particularly important to deal with metal fracture failure problems.

The working environment of titanium alloy components is often complex, e.g., aircraft blades, submersibles, and ships. The possibility of local damage to titanium alloy components increases greatly under complex working conditions, such as fatigue, corrosion, high temperature, stretch, and torsion, and these injuries are often difficult to determine and repair in the early stage of formation. After continuous accumulation and expansion, crack initiation and even failure of titanium alloy components can occur, seriously threatening the safety of personnel and equipment [10,11]. Therefore, studying the damage evolution and failure mechanism of titanium alloys is important for safe usage of equipment and widening the application of titanium alloys.

Fracture failure of materials is an extremely complex micromechanical process [12,13,14]. In numerical simulation, the selection of accurate material damage models and damage parameters is very important for reasonable accuracy of the calculation results. Many damage models are currently available, among which the metal fracture failure criteria commonly used in general nonlinear software are the shear damage criterion, flexible damage criterion, maximum shear stress criterion, Johnson–Cook damage model, FLD damage criterion, and M-K damage criterion [15,16,17]. For ductile metal damage, shear damage is used to predict the damage caused by shear band localization, while the FLD and M-K damage criteria are used to predict the damage caused by metal sheet formation. As the Johnson–Cook damage model takes into account the effects of stress triaxiality, strain rate, and temperature, the model parameters have clear meanings and are easy to determine through experiments; thus, this model is widely used in the finite element study of many material failures [18].

Many people have studied the Johnson-Cook damage model and metal fracture behavior in the past. Vasu et al. [19] used the Johnson–Cook damage model to conduct orthogonal cutting simulation research on Al7075-T6 material, and the predicted chip thickness was in good agreement with the experimental results. Careful experimental measurement of the Johnson–Cook damage parameters is an important prerequisite for obtaining good prediction results. Hu et al. [20] determined the Johnson–Cook damage model parameters of 45CrNiMoVA material through experiments and verified the validity of the parameters through comparative SHTB experiments. Liu et al. [21] used two sets of Johnson–Cook models with different parameters to describe the constitutive relationships of GH4169 alloy in different temperature ranges and simulated the process of projectiles impacting the target plate via finite element simulation. They showed that the predicted ballistic limit and failure model were in good agreement with the experimental results. Wang et al. [22] proposed a modified Johnson–Cook constitutive model and a failure model to characterize the dynamic mechanical behavior of GH3536 superalloy commonly used in engine containment rings. They analyzed the impact resistance of the material’s honeycomb structure at different temperatures, impact velocities, and impact angles through numerical simulation. They showed that the modified model had more accurate characterization ability than the original J–C model. Zhang et al. [23] studied the dynamic mechanical properties of a new titanium alloy (TC4 T) and established a simplified Johnson–Cook model with failure criteria. They also verified the accuracy of the model through experiments and simulations. They established a three-dimensional finite element model of an aero-engine fan blade on the basis of the proposed Johnson–Cook model. Ugodilinwa et al. [24] studied the quasi-static and high-strain-rate compressive deformation behavior of the new aerospace superalloy Haynes282 under three different human treatment conditions. They also established Arrhenius and improved Johnson–Cook constitutive models to describe the dynamic mechanical behavior of the material at high strain rates and high temperatures. Bora et al. [25] and Wang et al. [26] introduced different expressions of Lode strain parameters into the Johnson–Cook failure model to characterize the dynamic failure behavior of materials. Wang et al. [27] measured a high-temperature constitutive model of a TC4 titanium alloy sheet and found that both the strain-compensated Arrhenius model and the modified J–C model showed good prediction accuracy. Through microscopic analysis, it was found that high temperatures and low strain rates contributed to increasing the number and depth of dimples. Hu et al. [28] studied the material properties of TC4 titanium alloy and 7075 aluminum alloy under uniaxial tension and compression behavior for typical aerospace alloys. The results showed that the mechanical properties of TC4 were better than those of 7070 aluminum alloy at the same tensile rate. At the same time, the combined power-law constitutive model was fitted. Wang et al. [29] studied the effects of thickness on the properties of a TC4 titanium alloy sheet from the perspectives of overall deformation, fracture morphology, and crack propagation. The results showed that the TC4 titanium alloy sheet exhibited a ductile–brittle transition in the thickness range of 0.6–1.8 mm. Wang et al. [30] studied the effect of notch depth on the fracture behavior of a TC4 titanium alloy sheet. The results showed that TC4 titanium alloy undergoes ductile–brittle transition with the increase in notch depth. For TC4 titanium alloy sheets, the notch can be regarded as a crack at a certain notch depth.

Although many scholars have done a lot of research on parameter acquisition and verification of Johnson-Cook constitutive model for various materials, there is still a lack of description of the Johnson–Cook damage model of TC4 titanium alloy at room temperature and the experimental determination of the related parameters. In this study, TC4 titanium alloy was taken as the research object. The stress–strain curves at different strain rates were obtained by tensile testing at room temperature, and a Johnson–Cook constitutive model was obtained by fitting. The relationships between the parameters of TC4 titanium alloy based on the Johnson–Cook fracture failure model were studied by tensile testing of different notched specimens combined with finite element simulation. The fracture of TC4 was observed and analyzed by scanning electron microscopy, and the deformation mechanism of the TC4 titanium alloy was studied, providing a theoretical basis for predicting the fracture behavior of TC4 titanium alloy under tensile stress.

## 2. Mechanical Property Testing of TC4 Titanium Alloy

### 2.1. Material Selection

A TC4 titanium alloy sheet was used as the test sample. The raw material was composed of Ti-6Al-4V, and the chemical composition is shown in Table 1. All test samples were extracted from the same metal sheet using wire-cutting technology, and the thickness of the sheet was 2 mm.

### 2.2. Quasi-Static Tensile Test at Room Temperature

**Figure 1:**
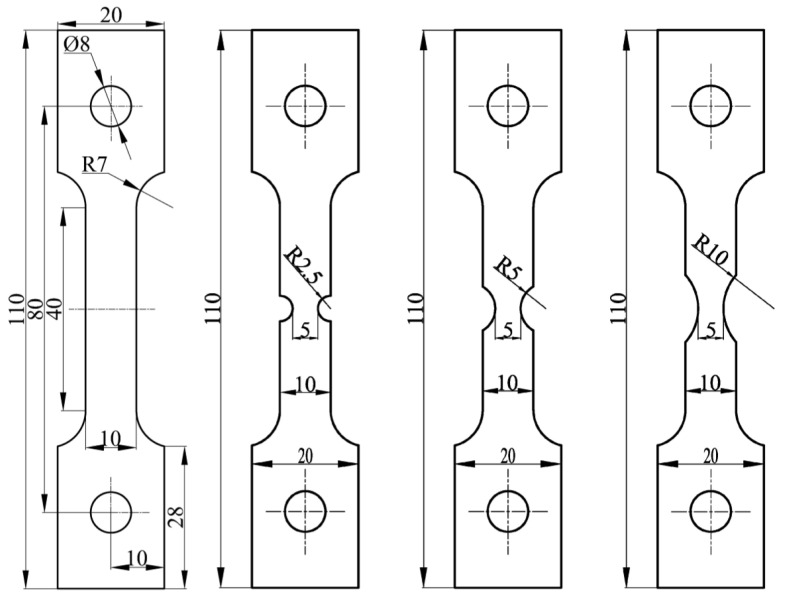
Tensile test specimen (unit/mm in figure).

Specimens with different notch radii were designed to determine the relationships between different stress triaxialities and fracture strains, as shown in Figure 1.

**Figure 2:**
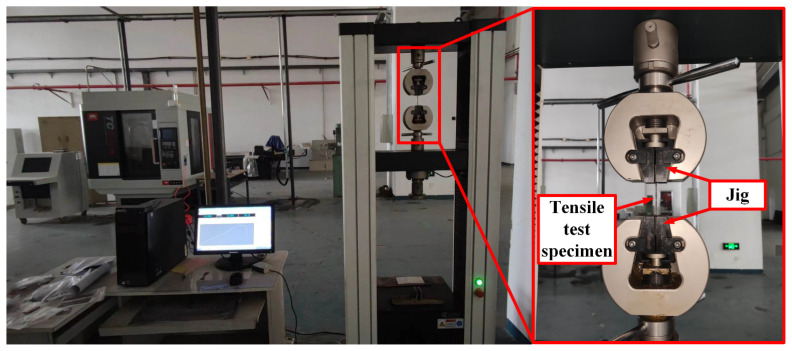
Universal material testing machine.

At room temperature, the quasi-static tensile test was carried out on a YSH-229WJ-10T microcomputer-controlled electronic universal material testing machine (Jinan Wance Electrical Equipment Co. Ltd, Jinan, China). The specimen mounting is shown in Figure 2. The test standard was based on ASTM E8 “Experimental method for tensile test of metal materials”. First, the specimen was preloaded with a failure load of no more than 30% of the expected failure load, and the tensile test was carried out after ensuring that no abnormalities were present. The quasi-static tensile load was applied to the specimen at different strain rates until the specimen was destroyed or the load decreased by 40% from the maximum value. The loading was stopped, and the maximum load and failure mode were recorded. Each group of experiments was carried out three times, and the average values were selected as the final data.

### 2.3. Dynamic Mechanical Test

**Figure 3:**
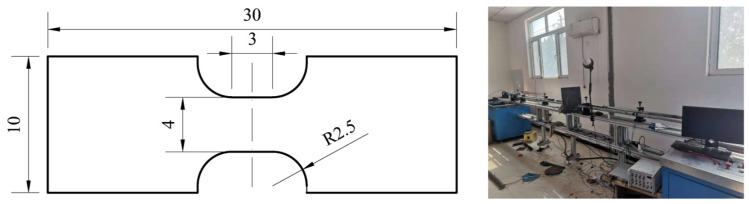
Split hopkinson tensile bar (unit/mm in figure).

Servohydraulic high-speed tensile testing machines and split Hopkinson tensile bars (SHTBs) are special equipment for dynamic tensile tests. In this paper, the dynamic mechanical behavior of a TC4 titanium alloy plate was studied using an SHTB. The engineering stress–strain curves of the TC4 titanium alloy at strain rates of 500 and 1000 s^−1^ were obtained. The test specimens and equipment are shown in Figure 3.

### 2.4. Dispose of Experimental Data

**Figure 4:**
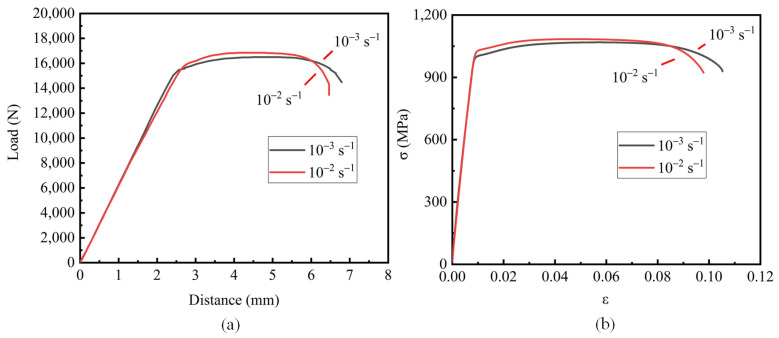
Smooth sample test data: (**a**) force–displacement curve; (**b**) stress–strain curve.

The load–displacement curve and the engineering stress–strain curve obtained from the quasi-static tensile test are shown in Figure 4.

**Figure 5:**
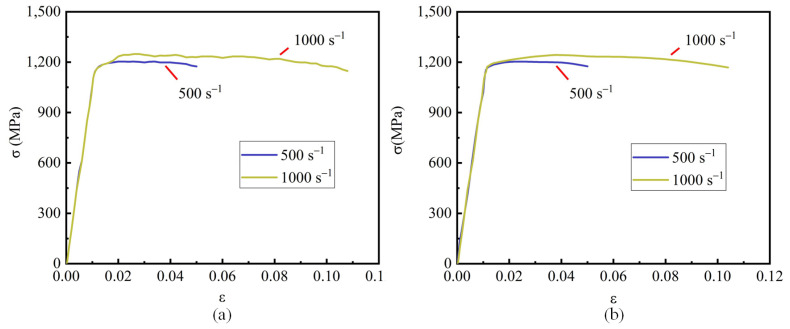
Dynamic tensile stress–strain curve of the smooth specimen: (**a**) experimental data; (**b**) smoothed experimental data.

The stress–strain curve obtained from the dynamic tensile test is shown in Figure 5a. Due to the oscillation of high-strain experimental data, the data were smoothed to facilitate the estimation of the material parameters, as shown in Figure 5b.

The experimental curve shows that the strain rate has no obvious effect on the flow stress at room temperature. Under the condition of constant volume, the engineering stress–strain curve of the material has the following relationship with the real stress–strain curve:

(1) $$\sigma_{t}=\sigma \left(1+\varepsilon \right)$$

(2) $$\varepsilon_{t}=\ln\left(1+\varepsilon \right),$$

where $\;\sigma_{t}$ is the true stress, $\varepsilon_{t}$ is the true strain, σ is the engineering stress, and ε is the engineering strain.

**Figure 6:**
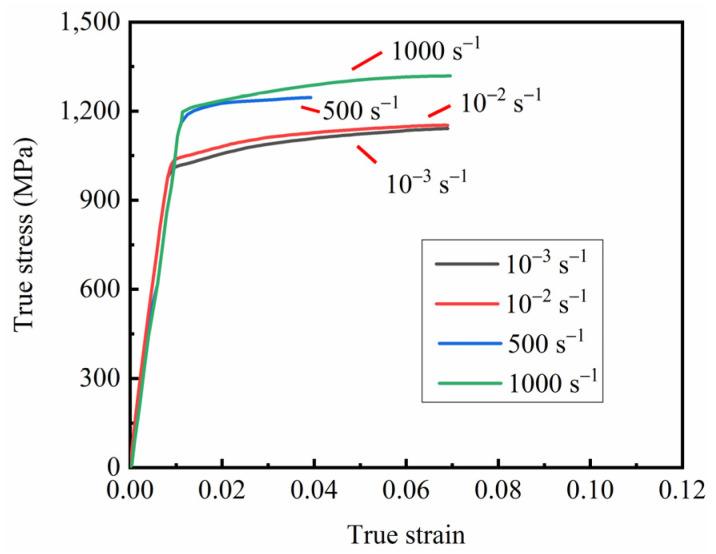
True stress–true strain curve of the smooth specimen.

The calculated true stress–true strain curve is shown in Figure 6.

## 3. Johnson–Cook Constitutive Model and Parameter Fitting

### 3.1. Johnson–Cook Constitutive Model

The material constitutive model needs to relate the flow stress with strain, strain rate, and temperature. Material models generally include the Johnson–Cook material model, Zerilli–Armstrong model, Steinberg–Guinan model, and Cowper–Symonds model [31,32,33,34]. The Johnson–Cook model is an empirical constitutive model proposed by Johnson and Cook in 1983 to describe the strain-rate-strengthening and thermal softening effects of metals [35,36,37]. They established an empirical yield function to describe plastic flow under dynamic loading, as follows:

(3) $$\sigma_{y}=\left(A+B\varepsilon_{p}^{n}\right)\left(1+C\ln{\dot{\varepsilon }}^{*}\right)\left(1-T^{*m}\right),$$

where *A*, *B*, *n*, *C,* and m are the material parameters; $\varepsilon_{p}$ is the equivalent plastic strain; ${\dot{\varepsilon }}^{*}={\dot{\varepsilon }}_{p}/{\dot{\varepsilon }}_{0}$ is the dimensionless equivalent plastic strain rate; ${\dot{\varepsilon }}_{p}$ is the strain rate;$\;{\dot{\varepsilon }}_{0}$ is the reference strain rate; and $T^{*}=\left(T-T_{r}\right)/\left(T_{m}-T_{r}\right)$ is the dimensionless temperature, where $T_{r}$ is the reference temperature (generally room temperature) and $T_{m}$ is the melting point of the material.

### 3.2. Johnson–Cook Failure Model

In 1985, Johnson and Cook [18] established the failure strain function of the empirical yield function as follows:

(4) $$\varepsilon_{f}=\left(D_{1}+D_{2}\exp D_{3}\sigma^{*}\right)\left(1+D_{4}\ln{\dot{\varepsilon }}^{*}\right)\left(1+D_{5}T^{*}\right),$$

where $\varepsilon_{f}$ is the failure plastic strain; $D_{1}-D_{5}$ are the failure model parameters; $\sigma^{*}$ is the stress triaxiality, defined as $\sigma^{*}=\sigma_{m}/\sigma_{eq}$, where hydrostatic pressure $\sigma_{m}=\left(\sigma_{11}+\sigma_{22}+\sigma_{33}\right)/3$,$\;\sigma_{eq}$ is the equivalent stress; ${\dot{\varepsilon }}^{*}$ is the dimensionless equivalent plastic strain rate; and $T^{*}$ is the dimensionless temperature.

Considering the changes in stress state, strain rate, and temperature during dynamic failure, the failure of the material was judged by the following plastic strain accumulation criterion:

(5) $$D=\sum \frac{\Delta \sigma_{eq}}{\varepsilon_{f}},$$

where $\Delta \sigma_{eq}$ is the equivalent plastic strain increment of a time step, and D is the damage parameter; when the value of D reaches 1, the material fails.

### 3.3. Parameter Fitting

The key to establishing the Johnson–Cook constitutive model and failure model of TC4 titanium alloy is to estimate the material parameters in the model in accordance with the existing experimental data. In this paper, the material constants of the TC4 titanium alloy were obtained in accordance with the stepwise estimation method [38,39,40]. As the experimental data were measured at room temperature, Equations (3) and (4) could be simplified as follows without considering the thermal softening effect of the materials:

(6) $$\sigma_{y}=\left(A+B\varepsilon_{p}^{n}\right)\left(1+C\ln{\dot{\varepsilon }}^{*}\right)$$

(7) $$\varepsilon_{f}=\left(D_{1}+D_{2}\exp D_{3}\sigma^{*}\right)\left(1+D_{4}\ln{\dot{\varepsilon }}^{*}\right)$$

At room temperature, the influence of temperature was not considered, and the experimental strain rate was the same as the reference strain rate. Formula (6) could be simplified as follows:

(8) $$\sigma_{y}=A+B\varepsilon_{p}^{n}$$

The values of A, B, and n could be obtained by fitting the true stress–true strain curve of the smooth sample. When the temperature was room temperature, the two ends of Equation (6) were deformed to obtain the following:

(9) $$\frac{\sigma_{y}}{A+B\varepsilon_{p}^{n}}=1+Cln{\dot{\varepsilon }}^{*}$$

The parameter C could be obtained by fitting the true stress–true strain curve of the smooth specimen.

The Johnson–Cook constitutive model parameters of the TC4 titanium alloy are summarized in Table 2.

**Figure 7:**
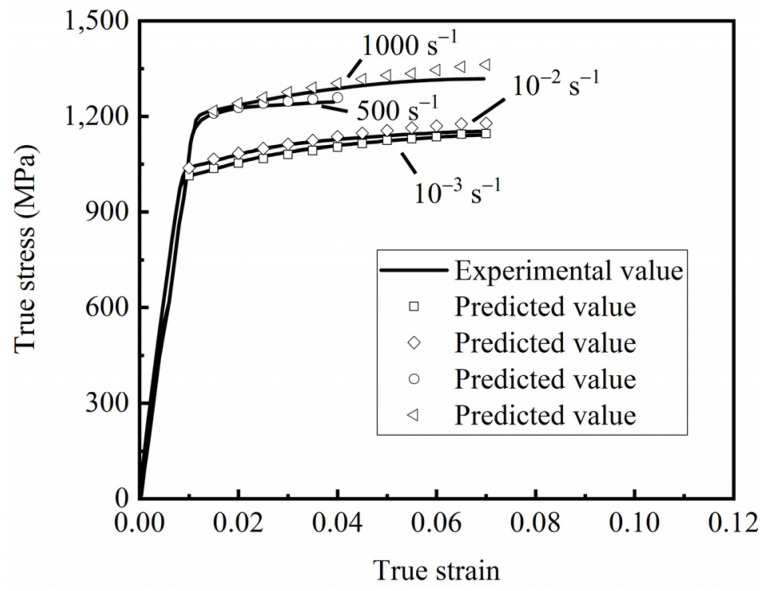
Relationship between experimental data and the Johnson–Cook model under different strain rates.

The comparison between the rheological stress predicted by the Johnson–Cook model at different strain rates and the experimental data is shown in Figure 7. The predicted values of the Johnson–Cook constitutive model provide a good fit for the experimental values. A reliable material constitutive equation was provided for the subsequent finite element simulations.

**Figure 8:**
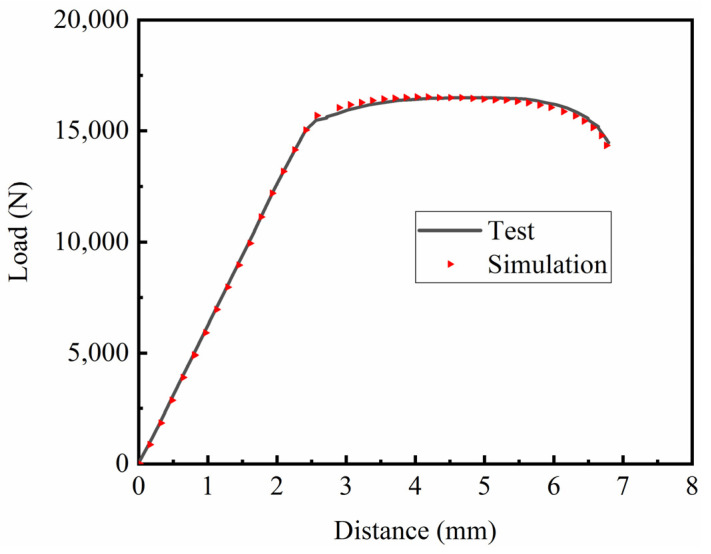
Comparison of the load–displacement curves of smooth specimens.

The fitting parameters of the TC4 titanium alloy’s elastic–plastic model were input into ABAQUS software 2020 for simulation calculations. The simulated strain rate was 10^−3^ s^−1^, and the load–displacement curve of the smooth specimen was obtained. The accuracy of the fitting parameters was determined by comparing the simulated load–displacement curve with the test load–displacement curve, as shown in Figure 8.

Given that ABAQUS did not show fracture behavior during the tensile process of the material (i.e., no fracture model parameters were inputted), after the parameters of the Johnson–Cook constitutive model were determined, the fracture displacement of the material’s fracture point during the test was used as the fracture displacement during the simulation. Then, the stress triaxiality $\sigma^{*}$ and the equivalent plastic strain $\varepsilon_{eq}\;$ of the material when the fracture occurred were determined, as shown in Table 3. Each stress triaxiality of the fracture failure criterion corresponded to a fixed fracture’s equivalent plastic strain. In the actual deformation process, the stress triaxiality of the material particles was constantly changing due to the different shapes of the samples, rather than a fixed value. To solve this situation, the stress triaxiality was integrated along the historical path during data processing to obtain the stress triaxiality, and the fracture criterion of the triaxiality and equivalent plastic strain was established.

Under the condition of the reference strain rate at room temperature, Equation (7) could be simplified as follows:

(10) $$\varepsilon_{f}=\left[D_{1}+D_{2}\exp D_{3}\sigma^{*}\right]$$

**Figure 9:**
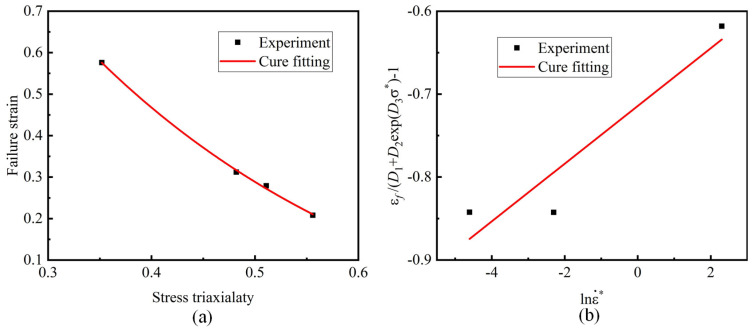
Johnson–Cook fracture model fitting curves: (**a**) fitting parameters *D*_1_–*D*_3_; (**b**) fitting parameter *D*_4_.

As shown in Figure 9a, the parameters *D*_1_–*D*_3_ were obtained by fitting the stress triaxiality and fracture strain of the material. The fracture strain of the material also decreased continuously with the increase in stress triaxiality.

The two ends of Equation (7) were deformed to obtain Equation (11). As shown in Figure 9b, Equation (11) is linearly fitted, and the slope is *D*_4_.

(11) $$\frac{\varepsilon_{f}}{D_{1}+D_{2}\exp D_{3}\sigma^{*}}-1=D_{4}\ln{\dot{\varepsilon }}^{*}$$

The fitted Johnson–Cook failure model parameters of the TC4 titanium alloy are shown in Table 4.

## 4. Result and Discussion

### 4.1. ABAQUS Simulation Verification

**Figure 10:**
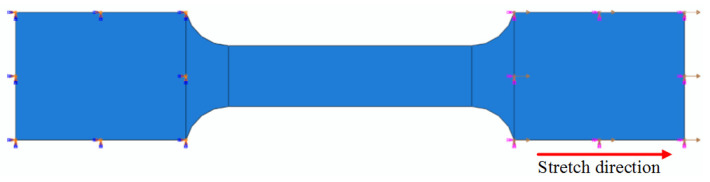
ABAQUS simulation model’s boundary conditions.

By using the established Johnson–Cook constitutive model and fracture failure model, the finite element software ABAQUS was used to recalculate all of the samples, and the ABAQUS calculation results were compared with the test results to verify the accuracy of the failure model. The boundary conditions of the model are shown in Figure 10. The boundary conditions of the sample were applied in accordance with the test loading direction, and the simulated strain rate was consistent with the test strain rate, which was the reference strain rate. The left end of the model was fully constrained, and the right end was free in the tensile direction, applying the same tensile speed as the test to ensure a consistent strain rate.

**Figure 11:**
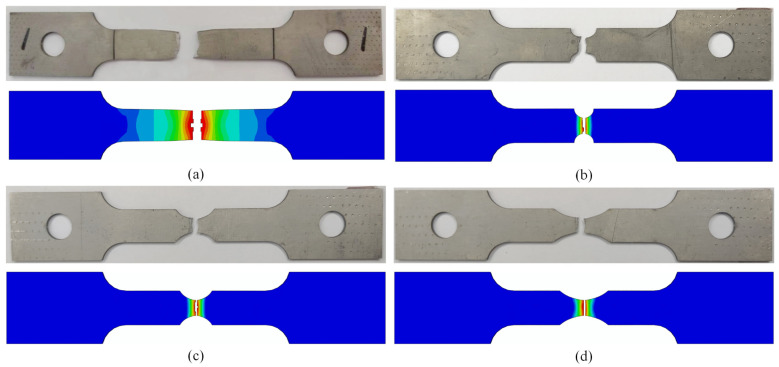
Comparison of tensile test and ABAQUS simulation fracture morphology: (**a**) smooth specimen; (**b**) notch 1 specimen (R = 2.5 mm); (**c**) notch 2 specimen (R = 5 mm); (**d**) notch 3 specimen (R = 10 mm).

All of the samples were simulated by ABAQUS, and the strain rate was selected as 0.001 s^−1^. The simulation results were compared with the experimental results, as shown in Figure 11. The results showed that the trend of the simulated fracture was essentially the same as that of the test fracture, and the simulation and the test exhibited a certain necking phenomenon at the fracture position.

**Figure 12:**
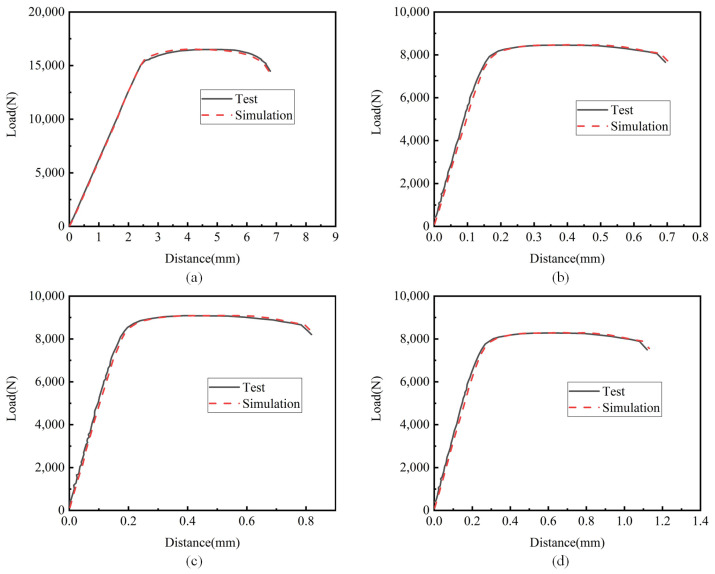
Force–displacement curve comparison diagram: (**a**) smooth specimen; (**b**) notch 1 specimen (R = 2.5 mm); (**c**) notch 2 specimen (R = 5 mm); (**d**) notch 3 specimen (R = 10 mm).

The load–displacement curve obtained by simulation was compared with the load–displacement curve obtained from the test, as shown in Figure 12, which shows the load–displacement curve under different notches. The curves obtained by simulation and the test coincided well, further confirming the reliability of the Johnson–Cook constitutive model parameters and damage parameters of TC4 titanium alloy fitted in this paper at room temperature.

### 4.2. Tensile Simulation Results

**Figure 13:**
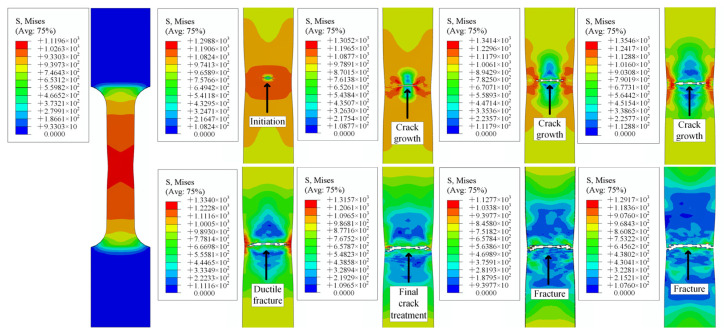
ABAQUS simulation of crack behavior.

**Figure 14:**
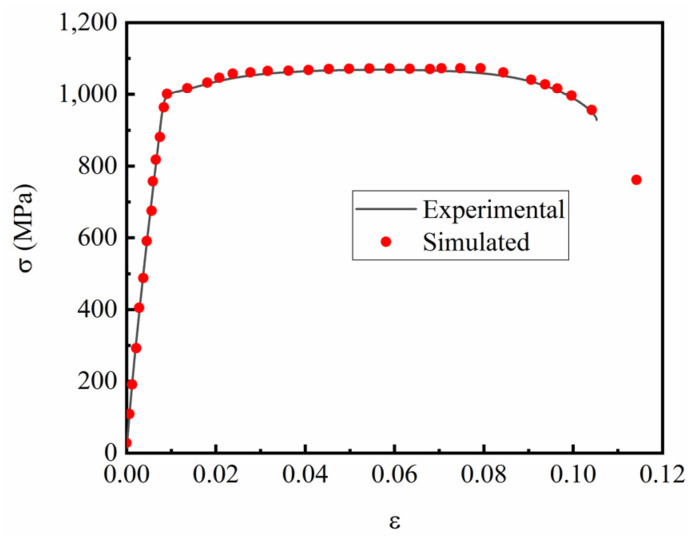
Comparison of stress–strain curves from the test and simulation.

Tensile simulation of smooth specimens was carried out using ABAQUS, as shown in Figure 13. The tensile specimen showed an obvious necking phenomenon before fracture. The enlarged diagram shows the crack initiation, propagation, and ductile fracture of the TC4 titanium alloy’s gauge section. At the beginning of alloy necking, microvoids and interfaces are known to accumulate near secondary-phase particles or inclusions, resulting in small cracks. In addition, as the plastic strain increases, these cracks propagate and are mainly responsible for weakening the specimen in the center of the necking zone. This phenomenon was well captured in the ABAQUS simulation work. The enlarged image in Figure 13 marks the stages of crack propagation and ductile fracture with arrows. These results clearly indicate that, in the tensile curve, plasticity is compatible with plastic strain. From the enlarged view of the tensile failure specimens, this ductile tearing phenomenon is also obvious. In addition, the stress–strain curve comparison diagram was tracked, as shown in Figure 14. The simulation curves were found to be in good agreement with the experimental results.

**Figure 15:**
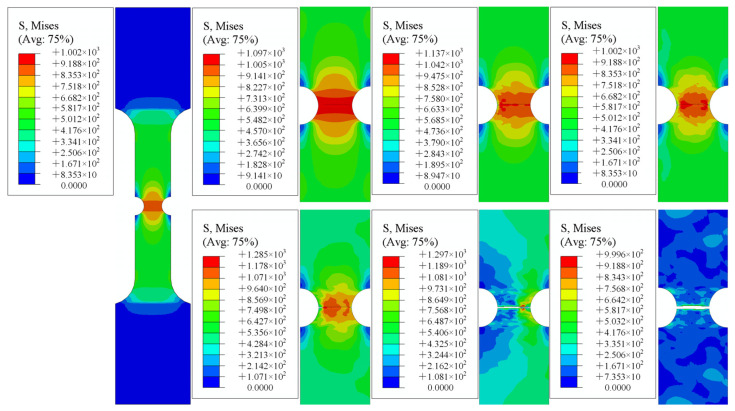
Notch specimen 1 (R = 2.5 mm).

**Figure 16:**
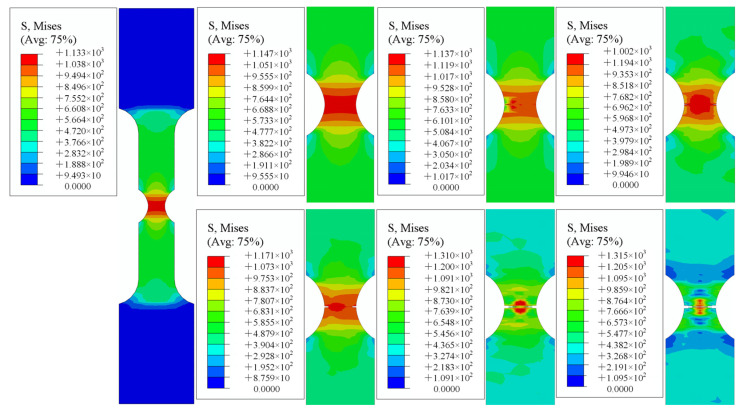
Notch specimen 2 (R = 5 mm).

**Figure 17:**
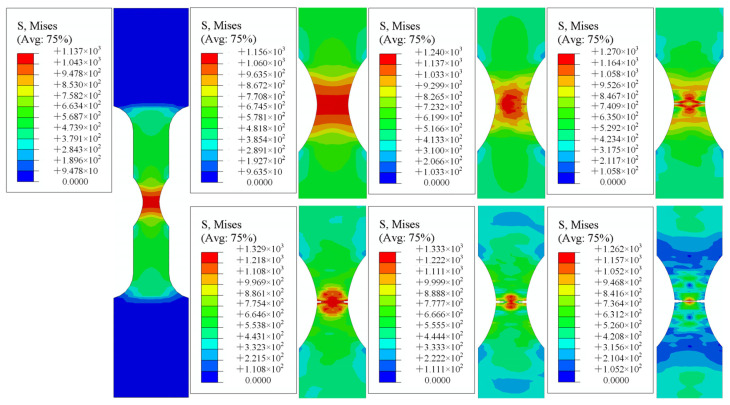
Notch specimen 3 (R = 10 mm).

Figure 15, Figure 16 and Figure 17 demonstrate the numerical tensile simulation processes of samples with different notches. The enlarged images show the process from the initiation, to the extension of the crack, to the fracture of the sample. The notched sample simulation differed from the smooth sample simulation in that the initial cracks of all notched samples were generated on both sides of the sample, whereas the cracks of the smooth samples were generated in the central part at the beginning. As the path on both sides of the notch specimen was not the same as that of the smooth specimen, both sides of the notch could be subjected not only to the force perpendicular to the direction of the section, but also to the force on the path on both sides of the notch during the tensile process of the specimen. The resultant force on both sides was greater than the force on the center of the specimen, so cracks could start from both sides of the specimen.

### 4.3. Tensile Fracture Morphology Analysis

In the tensile tests, fracture generally occurred in places where the structure was relatively weak, and the fracture could well reflect the cause, nature, mode, and mechanism of the alloy fracture. For enhanced understanding of the changes in the mechanical properties of TC4 titanium alloy, the fracture morphology of the tensile test sample was measured and analyzed.

**Figure 18:**
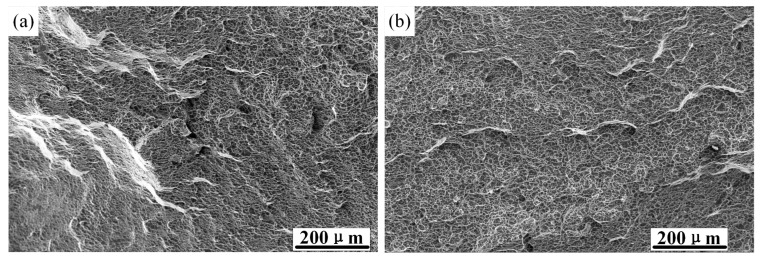
Tensile fracture morphology of a smooth specimen: (**a**) tensile fracture morphology at a test strain rate of 0.001 s^−1^; (**b**) tensile fracture morphology at a test strain rate of 0.01 s^−1^.

The tensile fracture morphology photographed by scanning electron microscopy is shown in Figure 18. The tensile fracture of TC4 titanium alloy belongs to dimple fracture. Figure 18a,b show the tensile fracture morphologies of smooth specimens at strain rates of 0.001 and 0.01 s^−1^, respectively. The fracture morphology depended on the microstructure, and shallow dimples and pores could be observed.

**Figure 19:**
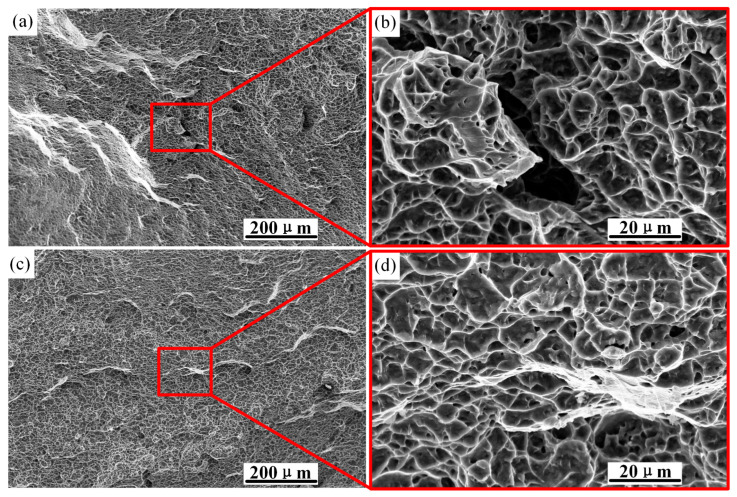
Fracture morphology of smooth specimens at 1000 and 4000 magnification: (**a**) strain rate 0.001 s^−1^; (**b**) local magnification; (**c**) strain rate 0.01 s^−1^; (**d**) local magnification.

Figure 19 shows the fracture morphology of the smooth specimen at 500 and 4000x magnification. It was covered with dimples of different sizes, which were caused by the initiation and coalescence of micropores. Obvious traces of plastic deformation could also be observed, and some areas clearly showed characteristics of metal tearing. Figure 19b,d show partial enlarged views at strain rates of 0.001 s^−1^ and 0.01 s^−1^, respectively. It is obvious that the dimples in Figure 19b are deep and dense. The tensile strength and elongation of the material at different strain rates obtained by tensile tests of the smooth specimen are shown in Table 5. It can be seen that with the increase in strain rate, the tensile strength of the material increases, while the elongation after fracture is the opposite, and the increase in strength is accompanied by a decrease in ductility. Therefore, high density and deep dimples represent high elongation.

During the tensile process of the sample, dislocation slip led to plastic deformation of the crystal. Since the stress distribution was not uniform at the microscopic scale, local concentrated stress was easily generated at the interfaces of the α/β-phase interface and the grain boundary, where dislocations accumulated. Macroscopically concentrated stress caused fatigue cracks in the object, while microscopically concentrated stress led to a weak interface. When the concentrated stress exceeded the interface binding force and the dislocations accumulated at the α/β boundary, microvoids were formed at the α-phase boundary. When dislocations accumulated inside the α-phase, microcracks appeared inside the α-phase. At this time, the grain boundary cluster α-phase could not hinder the movement of the dislocations, so deformation and dislocations gathered at the β-grain boundary and, finally, cracks extending along the grain boundary were formed in the β-grain boundary. With the continuous tensile test, the alloy broke due to the connection of these microcracks and microholes.

In Figure 19, there are a large number of dimples in the fracture morphology, indicating that the material has undergone a ductile fracture. The fracture mechanism is a mixed fracture mode caused by local necking and microvoid coalescence. In the experiment, when the strain reached a certain value, the microholes at the fracture grew and connected with the adjacent holes to form dimples.

## 5. Conclusions

In this paper, the Johnson–Cook constitutive model and failure model were simplified without considering the influence of temperature on the basis of the tensile testing of TC4 titanium alloy at room temperature. The Johnson–Cook constitutive model parameters and failure parameters of the TC4 titanium alloy were also obtained by fitting. The accuracy of the constitutive model and the failure model was verified by tensile test simulations. Through the simulation of the smooth sample, the whole process of the sample—from crack initiation to fracture—was observed, and the tensile fracture was analyzed by scanning electron microscopy. The following conclusions could be drawn:

1. The tensile test of TC4 titanium alloy at different strain rates at room temperature was carried out. The Johnson–Cook constitutive model parameters and failure parameters were obtained by fitting. Through verification, the predicted results of the constitutive model were found to be close to the experimental results. The constitutive model exhibited good accuracy, providing a theoretical basis for the tensile simulation of TC4 titanium alloy.
2. Through the finite element simulation of the test specimen, the whole process from crack initiation to specimen fracture was obtained. The crack in the smooth specimen was first generated in the center. With continuous accumulation of plastic strain, the crack expanded to both sides until the specimen was broken. The cracks in notched specimens were generated from both sides and extended to the middle until the specimens were broken.
3. The tensile fracture of TC4 titanium alloy belongs to dimple fracture. When the tensile strain rate was low, the dimples were deep and dense. Compared with the experimental results, high density and deep dimples represented high elongation.

## Figures and Tables

**Table 1:** Chemical composition of the TC4 titanium alloy.

Element	Al	V	Fe	C	N	H	O	Ti
Weight %	5.50–6.75	3.50–4.50	0.30	0.10	0.05	0.015	0.20	Balanced

**Table 2:** Johnson–Cook constitutive model parameters of the TC4 titanium alloy.

Constitutive Parameter	A (MPa)	B (MPa)	n	C
Stepwise estimation method	1003.132	1003.510	0.663	0.0137

**Table 3:** Stress triaxiality and equivalent plastic strain of each specimen.

Specimen Type	Stress Triaxiality	Breaking Strain
Smooth specimens	0.352	0.576
Notch specimen 1	0.556	0.208
Notch specimen 2	0.511	0.279
Notch specimen 3	0.482	0.312

**Table 4:** Johnson–Cook failure model parameters of the TC4 titanium alloy.

Model Parameters	*D*_1_	*D*_2_	*D*_3_	*D*_4_
Fitted value	−0.197	2.332	3.138	0.034

**Table 5:** Smooth experimental sample tensile data.

Tensile Strain Rate (s^−1^)	Tensile Strength (MPa)	Percentage Elongation after Fracture (%)
0.001	1069	16.5
0.01	1084	14
